# The Effects of Aging, Malingering, and Traumatic Brain Injury on Computerized Trail-Making Test Performance

**DOI:** 10.1371/journal.pone.0124345

**Published:** 2015-06-10

**Authors:** David L. Woods, John M. Wyma, Timothy J. Herron, E. William Yund

**Affiliations:** 1 Human Cognitive Neurophysiology Laboratory, Veterans Affairs Northern California Heath Care System, 150 Muir Rd., Martinez, CA, 95553, United States of America; 2 University of California Davis, Department of Neurology, 4860 Y St., Suite 3700, Sacramento, CA, 95817, United States of America; 3 Center for Neurosciences, University of California Davis, 1544 Newton Ct., Davis, CA, 95616, United States of America; 4 Center for Mind and Brain, University of California Davis, 202 Cousteau Place, Suite 201, Davis, CA, 95616, United States of America; University Of Cambridge, UNITED KINGDOM

## Abstract

The trail making test (TMT) is widely used to assess speed of processing and executive function. However, normative data sets gathered at different sites show significant inconsistencies. Here, we describe a computerized version of the TMT (C-TMT) that increases the precision and replicability of the TMT by permitting a segment-by-segment analysis of performance and separate analyses of dwell-time, move-time, and error time. Experiment 1 examined 165 subjects of various ages and found that completion times on both the C-TMT-A (where subjects connect successively numbered circles) and the C-TMT-B (where subjects connect circles containing alternating letters and numbers) were strongly influenced by age. Experiment 2 examined 50 subjects who underwent three test sessions. The results of the first test session were well fit by the normative data gathered in Experiment 1. Sessions 2 and 3 demonstrated significant learning effects, particularly on the C-TMT-B, and showed good test-retest reliability. Experiment 3 examined performance in subjects instructed to feign symptoms of traumatic brain injury: 44% of subjects produced abnormal completion times on the C-TMT-A, and 18% on the C-TMT-B. Malingering subjects could be distinguished from abnormally slow controls based on (1) disproportionate increases in dwell-time on the C-TMT-A, and (2) greater deficits on the C-TMT-A than on the C-TMT-B. Experiment 4 examined the performance of 28 patients with traumatic brain injury: C-TMT-B completion times were slowed, and TBI patients showed reduced movement velocities on both tests. The C-TMT improves the reliability and sensitivity of the trail making test of processing speed and executive function.

## General Introduction

The trail making test (TMT) is the third most widely used test in neuropsychology [1] and has been incorporated into a number of assessment batteries, including the Halstead-Reitan battery [2] and the Delis-Kaplan executive function system [3]. The standard TMT comes in two forms: Trails A, where subjects connect a series of 25 numbered circles in ascending order, and Trails B, where subjects connect 25 circles alternating between ascending numbers and letters (e.g., 1-A-2-B, etc.). Completion times on the TMT are used to assess visual attention, speed of processing, mental flexibility, and executive function in patients by comparisons with normative data from appropriate control populations [4].

However, TMT norms show considerable unexplained variability [5]. Table 1 presents data norms collected in large-scale studies performed since 1998, and reveals large variations of average completion times in the norms for both Trails A (range 23.4 to 70.2 s) and Trails B (range 54.3 to 157.7 s). While some of these differences can be accounted for by the strong effects of age and education on completion times [4], differences remain among subject groups with similar demographic characteristics. For example, Ising, Mather [6] studied two groups of German subjects with similar mean ages (48.9 and 47.4 years) and years of education (10.5 and 10.6 years): Trails A completion times (25.7 vs. 30.0 s) differed by more than 0.5 standard deviation between the two groups [t(888) = 8.32, p < 0.0001]. Across-laboratory differences can be even more pronounced. For example, Poreh, Miller [7] and Perianez, Rios-Lago [8] studied subjects of similar mean ages (38.2 and 38.9) and relatively similar years of schooling (14.5 vs. 13.3 years), but found respective means that differed by nearly one standard deviation on Trails A [t(492) = 14.74, p < 0.0001], along with significant differences on Trails B [t(492) = 2.14, p < 0.02]. Even larger differences have been observed in TMT norms gathered in different countries [9, 10], among different ethnic groups [11], and even among NFL football players tested at different sites [12].

**Table 1:** Recent large scale studies of normative Trails A and B performance.

Study	N	Age	Range	Trail A	SD	Age Slope	Trail B	SD	Age Slope	B-A
**Atkinson et al. (2010)**	154	21.3	18–45	23.4	7.2		54.3	16.2		30.9
**Poreh et al. (2012)**	271	38.2	18–90	23.8	7.8	0.6	58.1	22.0	1.3	34.3
**Levine et al. (2007)**	344	41.4	20–60	25.4	8.5		55.7	20.3		30.2
**Ising et al. (2014)**	350	48.9	20–75	25.7	9.3		68.8	28.7		43.1
**Li et al. (2014)**	315	57.4	22–85	27.4	11.0	0.2	67.7	31.2	0.6	40.3
**Ising et al. (2014)**	540	47.4	18–80	30.0	10.9		73.0	34.9	-	43.0
**Perianez et al. (2007)**	223	38.9	16–80	31.7	13.7	0.3	68.1	43.2	1.0	36.4
**Heaton et al. (1999)**	74	68.2	60–80	34.8	12.4		98.5	49.1		63.8
**Ashendorf et al. (2008)**	269	72.4	55–90	35.7	12.8	0.5	81.5	36.1	1.4	45.8
**Tombaugh et al. (2004)**	878	57.6	18–84	37.3	12.5	0.6	87.5	28.0	1.6	50.1
**Schneider et al. (2014)**	392	72.4	65–80	39.3	14.0		107.3	44.8	-	68.0
**Ising et al. (2014)**	448	78.2	70–85	40.9	11.4		101.0	36.1	-	60.1
**Vazzana et al. (2010)**	583	72.5	65–78	70.2	29.6		157.7	62.0	-	87.5

Data are ordered by Trails A completion times. SD = standard deviation. Age-slope = increase in completion times in s/year, estimated from available data. B-A = difference between Trails B and Trails A completion times.

Since the traditional TMT test has a standard layout, the variability in TMT norms suggests that differences in test administration procedures may have a significant influence on TMT results [13]. The examiner measures TMT completion times with a stopwatch, with most examiners timing from the moment when the start command is given. In addition, the examiner must monitor the subject throughout the test to assure that they connect each circle [14]. In the event of an error, the examiner stops the subject, crosses out the erroneous connecting lines, and makes sure that the subject returns to the last correct circle. Error-correction time will vary for different examiners, as do other aspects of TMT administration. Examiners also differ in the stringency with which they enforce the requirement that connecting lines must enter each circle; some will accept connecting lines slightly outside circle boundaries, while others treat these as errors. In addition, examiners use different corrective procedures for other non-error conditions, such as changing the orientation of the paper, lifting the pencil from the page, or attempting to erase a response (e.g., some examiners remove the pencil’s eraser). Thus, TMT completion times will reflect not only the subject’s ability, but also the examiner’s timing, efficiency at correcting errors, and test administration procedures.

The comparison of completion times on Trails B and Trails A, using subtractions or ratio measures, also plays an important part in TMT interpretation [4]. While the commonly-used subtraction method is often assumed to reflect differences in the cognitive demands of the two tests, measurements show that Trails A and Trails B path lengths differ significantly. For example, in the common Reitan version of the TMT, the Trails B path (243 cm) is 32% longer than the Trails A path [15], while on the D-KEFS version of the TMT [16], paths in the alternating letter-number sequences (298 cm) are 24.4% shorter than the summed path lengths for letter-only and number-only tests. Thus, Trails B-A difference scores on both versions of the TMT may include the contribution of drawing speed in addition to contributions from other aspects of cognitive function.

Finally, while the same TMT display is seen by all subjects at the beginning of the test, the display changes throughout the test as a result of the lines drawn by the subject. In particular, subjects who draw imprecisely or make errors will add visual clutter, which complicates the detection of subsequent targets. As a consequence, the difficulty of later portions of the TMT will vary from subject-to-subject in a counter-adaptive manner: i.e., the test will become increasingly difficult for subjects with poorer performance, further amplifying abnormalities in completion times.

Here, we introduce a computerized version of the TMT (C-TMT) that systematizes TMT administration, reduces the influence of the examiner, automatically corrects errors, equates Trails A and Trails B path lengths, and presents a standardized TMT display throughout the test that is consistent across subjects. The C-TMT also permits a segment-by-segment analysis of performance and adds a number of new metrics that provide additional insight into the different factors contributing to overall completion time.

We describe four experiments using the C-TMT. In Experiment 1, we analyze data from 165 control subjects ranging in age from 18–84 years. Experiment 1 was used to evaluate the effects of age, sex, education, and computer-use on performance, and to extract predictive z-score norms in order to correct for these factors in Experiments 2–4. In Experiment 2, 50 naïve control subjects underwent three successive test sessions at weekly intervals. Session 1 was used to evaluate the goodness-of-fit of the regression functions obtained in Experiment 1, while sessions 2 and 3 permitted the analysis of test-retest reliability and learning effects. In Experiment 3, the subjects from Experiment 2 were instructed to feign symptoms of traumatic brain injury during a fourth test session. The goal of Experiment 3 was to quantify the effects of malingering on TMT completion times and, more importantly, to develop malingering indices that could assist in determining whether C-TMT abnormalities were due to suboptimal effort or organic causes. Finally, in Experiment 4, we examined the sensitivity of the C-TMT to cognitive deficits consequent to traumatic brain injury (TBI).

## Experiment 1: The Effects of Age, Education, Computer-Use, and Sex

Completion time on the TMT is the sum of the time needed to connect each of the 25 circles, plus any additional error time. The C-TMT measures error time and circle-connection time separately. The time needed to draw a line to the next circle reflects the *dwell-time*, the time needed to locate the next circle before drawing begins, and *move-time*, the time required to draw a line to the next circle and select it. Move-time, in turn, reflects drawing *velocity*, the speed of drawing, and line length or *circuitousness* (i.e., the ratio of the number of pixels drawn by the subject relative to the number of pixels in the shortest straight line connecting the two circles).

Two main factors were expected to influence the time required to complete different segments of the C-TMT. First, we expected to find variations in move-time that correspond to the varied lengths of different C-TMT segments. Second, as the test progressed, more circles were connected, so that the search for unconnected circles would be facilitated. Therefore, we expected that dwell-times would be reduced over the course of the test.

Age has a major influence on completion times (Table 1). Trails A completion times show age slopes ranging from 0.2 s/year [17] to 0.6 s/year [4, 7], while Trails B age slopes are steeper, ranging from 0.6 s/year [17] to 1.6 s/year [4]. Although the age-related changes are larger for Trails B than Trails A [10], the Trails B/A ratio is generally insensitive to age [18]. Given the abundant evidence of age-related motor slowing [19], we anticipated that age-related slowing would be due primarily to increased move-times and reductions in movement velocity.

Education also influences TMT performance [4, 16], although its influence is weaker than the effects of age [20]. Education effects are generally larger on Trails B than Trails A [4], and can be substantial in subjects with limited education [8, 21, 22]. For example, in some studies, the difference on Trails B completion times between subjects with a grade school and college education is equivalent to that associated with a 10–15 year difference in age [23, 24].

We also investigated two other demographic variables: computer-use and sex. We anticipated that subjects who used computers extensively would show increased drawing precision and speed. Sex, on the other hand, has inconsistent effects on TMT performance, with some studies reporting small male advantages [8, 21, 25, 26], while others report insignificant differences [4, 27] or faster completion times in women [23].

TMT error rates (i.e., connections to incorrect circles) are generally low, ranging from 0.0 to 0.3 on Trails A, and from 0.2 to 0.8 on Trails B [7, 28–30]. Error rates increase in older subjects [27], particularly on Trails B [29]. However, error time is not separately quantified in the traditional TMT. We anticipated that error rates would be somewhat higher on the C-TMT than on manually administered TMTs, because all errors, including failing to completely enter a circle, were consistently scored.

Finally, the different C-TMT metrics also enabled us to examine the factors that increase completion times on the C-TMT-B in comparison with the C-TMT-A. In the C-TMT-B, the complexity of target selection is increased (i.e., letters and numbers must be alternatively selected), so we hypothesized that dwell-time, as well as the dwell-time/total-time ratio, would increase in the C-TMT-B in comparison with the C-TMT-A.

### General methods

#### Ethics statement

Subjects in all experiments gave informed written consent following procedures approved by the Institutional Review Board of the VA Northern California Health Care System (VANCHCS) and were paid for their participation.

#### Apparatus

Testing was performed in a quiet testing room using a standard PC controlled by Presentation software (Versions 13 and 14, NeuroBehavioral Systems, Berkeley, CA). Subjects sat 0.7 m from a 17” Samsung Syncmaster LCD monitor, whose refresh rate was 60 Hz. Responses were recorded with a high-precision USB PC gaming mouse (Razer Sidewinder, Carlsbad, CA) sampled at 1.0 kHz. Button closure required a movement of 2.0 mm.

#### Procedure

C-TMT testing required about ten minutes and occurred midway through a two-hour test session that included computerized tests of finger tapping, simple reaction time, choice reaction time, Stroop interference, digit span forward and backward, verbal fluency, verbal list learning, spatial span, design fluency, the Wechsler Test of Adult Reading (WTAR), the Paced Auditory Serial Addition Test (PASAT), the Cognitive Failures Questionnaire (CFQ), the Posttraumatic Stress Disorder Checklist (PCL), and a traumatic brain injury (TBI) questionnaire.

**Fig 1:**
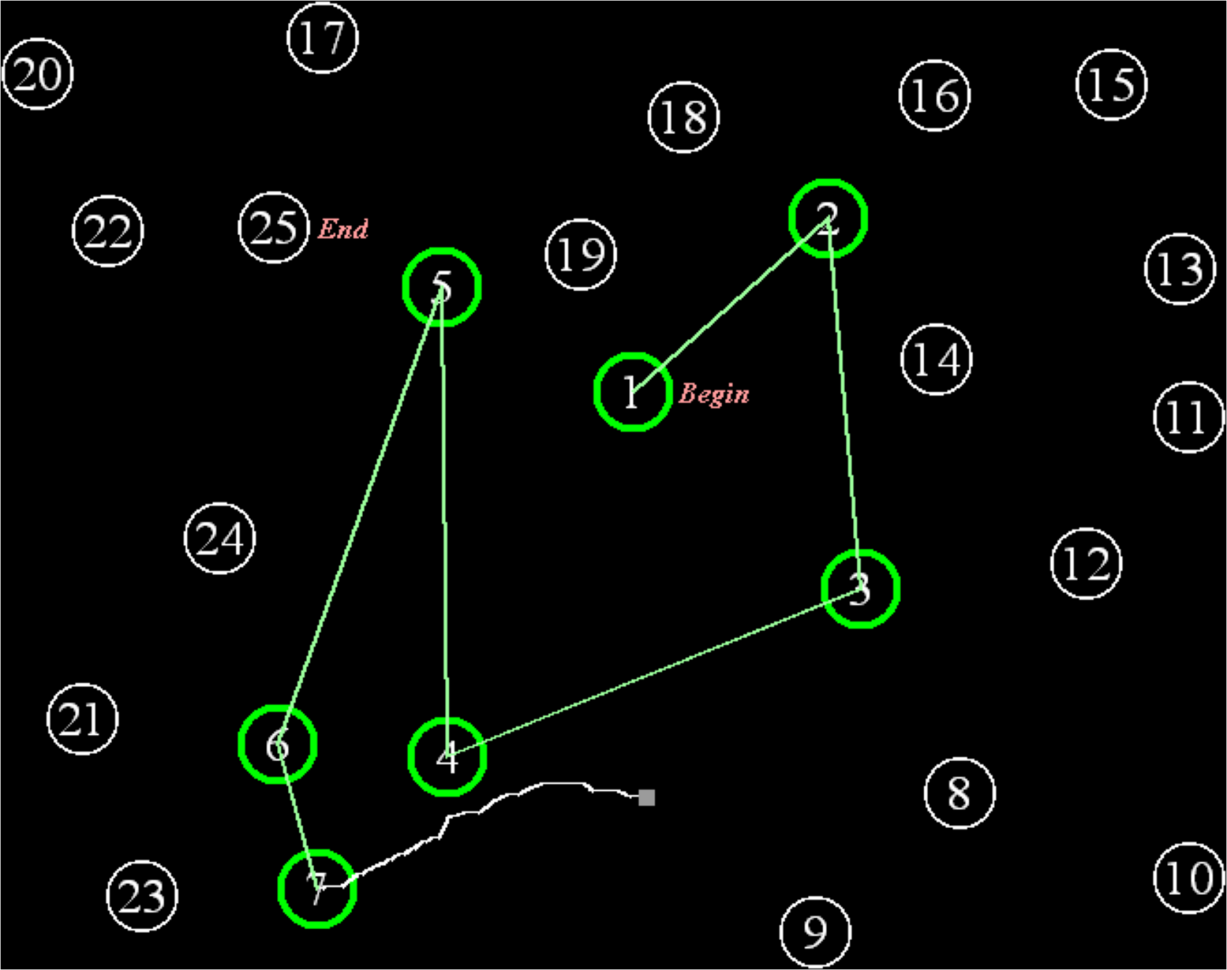
The C-TMT. The display as seen by a subject midway between connecting circles 7 and 8 on the C-TMT-A. As each circle was correctly connected, its color changed from white to green (e.g., circles 1–7). The current path between circles was shown in white (e.g., between 7 and 8), but was replaced by a straight green line as each segment was completed.

The C-TMT differed from the standard TMT in several ways (Fig 1). Subjects connected circles displayed on the computer monitor using the mouse. On each C-TMT segment, the path currently being drawn was displayed as a white line, and the selection of each circle required a mouse click within its boundaries. This contrasts with the manually administered TMT, where subjects can “draw through” successive circles without stopping, and assured that lines connecting successive circles fell within each circle’s circumference.

The C-TMT provided additional color cues to reduce the difficulty of the search for unconnected circles. Once a circle had been successfully selected, it changed color from white to green, and the white path drawn by the subject was replaced by a straight green line connecting the circles. Overall, completion times were divided into the time needed to successfully connect all of the circles and any additional delays introduced by errors. Errors were automatically corrected and the erroneous path was erased. This ensured that all subjects saw identical patterns on each segment of the test: i.e., the visual clutter associated with errors and imprecise drawing paths was eliminated.

Subjects performed four practice trials to familiarize themselves with the process of using the mouse to connect circles, rather than the single practice trial in the standard paper-and-pencil TMT. The first practice trial included the first seven circles as they appeared on the test. The displays on the other three practice trials were mirrored about the vertical, horizontal, and both axes. Cursor position was measured by the continuous sampling (1000 Hz) of mouse position using the computer gaming mouse. Movement paths were displayed on each video refresh and times were measured with the high-precision digital clock (100 kHz), beginning when the subject clicked on the first circle.

Errors, classified as button presses in an incorrect circle, resulted in the circle flashing red three times (200 ms duration flashes) at 300 ms intervals, followed by the immediate return of the cursor to the last correct circle and the deletion of the incorrectly drawn line. Button presses outside the circumference of a circle were ignored. The time required for each error (including the 1000 ms error cue) was quantified. As on the standard TMT, total time was the time required to correctly connect all circles, plus any additional time associated with errors.

#### Statistical analysis

Correlation analysis was used to analyze the effects of age, education, computer-use, and sex, and to develop normative regression functions. Pairwise effects were analyzed with Student’s t-tests, using a model that assumes unequal variance in the different subject groups when appropriate. Group comparisons were further analyzed using a multifactor mixed ANOVA. Separate ANOVAs were performed for age- and computer-use regressed z-scores (see below) for total completion time and movement velocity. Greenhouse-Geisser corrections of degrees of freedom were uniformly used in computing p values in order to correct for covariation within factors or interactions. Effect sizes are reported as partial ω^2^ values.

#### Subjects

We gathered normative data on 165 subjects who were recruited through advertisements on Craigslist and from existing control-subject populations. The demographic characteristics of the subjects are shown in Table 2. Subjects ranged in age from 18–82 years and had high levels of education (mean 2.6 years of college, with less than 2% failing to complete high school). Subjects were required to meet the following inclusion criteria: (a) fluency in the English language; (b) no current or prior history of psychiatric disease; (c) no current substance abuse; (d) no concurrent history of neurologic disease known to affect cognitive functioning; (e) on a stable dosage of any required medication; (f) auditory functioning sufficient to understand normal conversational speech and visual acuity normal or corrected to 20/40 or better. Thirteen control subjects were excluded: six because of a history of head injury, two due to software errors, one because of headache during the test, one because of missing demographic information, and three for failing to follow instructions on the C-TMT-A. Subjects were paid for their participation. Subject ethnicities were 64% Caucasian, 12% African American, 14% Asian, 10% Hispanic/Latino, 2% Hawaiian/Pacific Islander, 2% American Indian/Alaskan Native, and 4% “other.” Most of the subjects used computers frequently, with 92.7% using computers for at least 1 hour/day.

**Table 2:** Participant characteristics.

Experiment	Group	N	Ages (yrs)	Education (yrs)	C-use scale	Male (%)
**Exp. 1**	Control	165	18–82; 41.5 (21.5)	10–20; 14.6(2.2)	5.21	58%
**Exp. 2/3**	Control/Malinger	50	18–46; 26.5 (5.7)	12–18; 15.0 (2.0)	5.88	48%
**Exp.4**	mTBI	25	20–61; 34.1(11.5)	10–18; 13.6(5.8)	4.97	100%
	sTBI	4	35–57; 46.0(9.0)	12–16; 13.5(1.9)		75%
	E-TBI	2	43,50	12,18		100%

mTBI = mild TBI. sTBI = severe TBI. E-TBI = excluded TBI patients, two patients excluded for evidence of suboptimal effort. C-Use = computer use (hours/day). Range, mean, and variance are shown for age and education.

### Results: Experiment 1

#### C-TMT-A: analysis by segment

**Fig 2:**
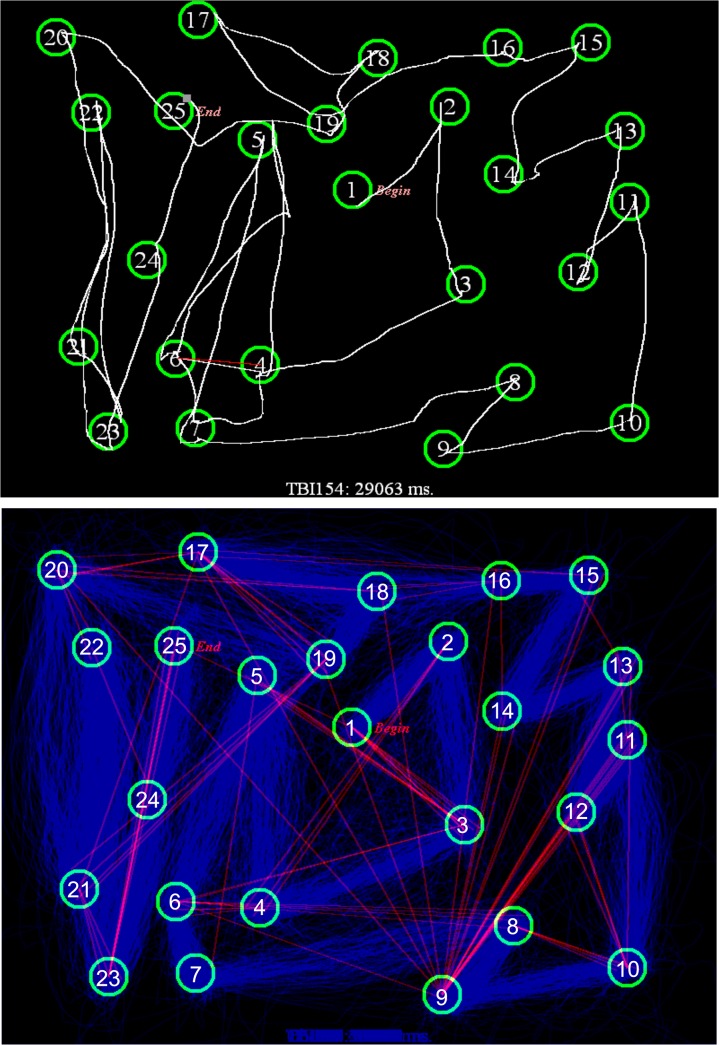
C-TMT-A paths. **A.** The path drawn by a subject on the C-TMT-A, containing an error shown by a red line connecting circles 4 to 6. The subject clicked outside circle 5, and then advanced to circle 6, which resulted in his return to circle 4 (white line). He then drew a circuitous connection to circle 5 through circle 7. No further errors were committed. **B.** The superimposed C-TMT-A paths of all Experiment 1 control subjects. Paths drawn by individual subjects are shown in blue. Erroneous connections are shown as straight red lines.

Fig 2A shows a bitmap of the C-TMT-A path drawn by a single subject, and Fig 2B shows the bitmaps of C-TMT-A paths for all subjects in Experiment 1, with erroneous connections displayed as straight red lines.

C-TMT-A path segments varied in length from 83 to 368 pixels. S1 Table (top) provides path lengths and average completion times (in ms) for each segment of the C-TMT-A and C-TMT-B, along with separate measures of dwell-time, move-time, circuitousness, and velocity for each segment. The correlation matrix for the different segments of the C-TMT-A is shown in Table 3 (top). As expected, longer segments required more time to connect [r = 0.49, t(22) = 2.64, p < 0.02], due primarily to increased move-time [r = 0.54, t(22) = 3.01, p < 0.01]. There was also the expected reduction in the dwell-time/total-time ratio with increasing path length [r = -0.55, t(22) = 3.09, p < 0.006]. Longer paths were associated with increased drawing velocity [r = 0.87, t(22) = 8.28, p < 0.0001], and velocity was negatively correlated with circuitousness [r = -0.73, t(22) = 5.01, p < 0.0001]; i.e., on longer segments, subjects drew straighter lines with greater speed. Completion times decreased over the successive segments of the C-TMT-A [r = -0.43, t(22) = -2.17, p < 0.05], and when the path length effect was parceled out, the correlation between segment number and completion time increased even further [r = -0.62, t(21) = -3.62, p < 0.002]. This was due primarily to reductions in dwell-time [r = -0.57, t(22) = -3.25, p < 0.005]; i.e., subjects were more easily able to detect the target circle.

**Table 3:** Pearson correlations between segment parameters and performance measures.

**Trails A**						
	**Length**	**Time**	**Dwell**	**Move**	**Circuit**	**Vel**
**N**	0.20	-0.43	-0.57	-0.36	-0.42	0.39
**Length**		0.49	0.29	0.54	-0.50	0.87
**Time**			0.93	0.99	0.33	0.12
**Dwell**				0.88	0.46	-0.02
**Move**					0.27	0.17
**Circuit**						-0.73
**Trails B**						
	**Length**	**Time**	**Dwell**	**Move**	**Circuit**	**Vel**
**N**	0.14	0.03	-0.23	0.15	0.18	0.08
**Length**		0.68	0.38	0.76	-0.36	0.72
**Time**			0.87	0.97	0.20	0.19
**Dwell**				0.72	0.31	0.03
**Move**					0.13	0.25
**Circuit**						-0.51

N = circle number. Dwell = dwell-time. Move = move-time. Circuit = circuitousness. Vel = velocity. Given the sample size (N = 24), correlations with |r| > 0.41 are statistically significant at the p < 0.05 level (uncorrected for multiple comparisons). Data from Experiment 1.

#### C-TMT-B: analysis by segment

**Fig 3:**
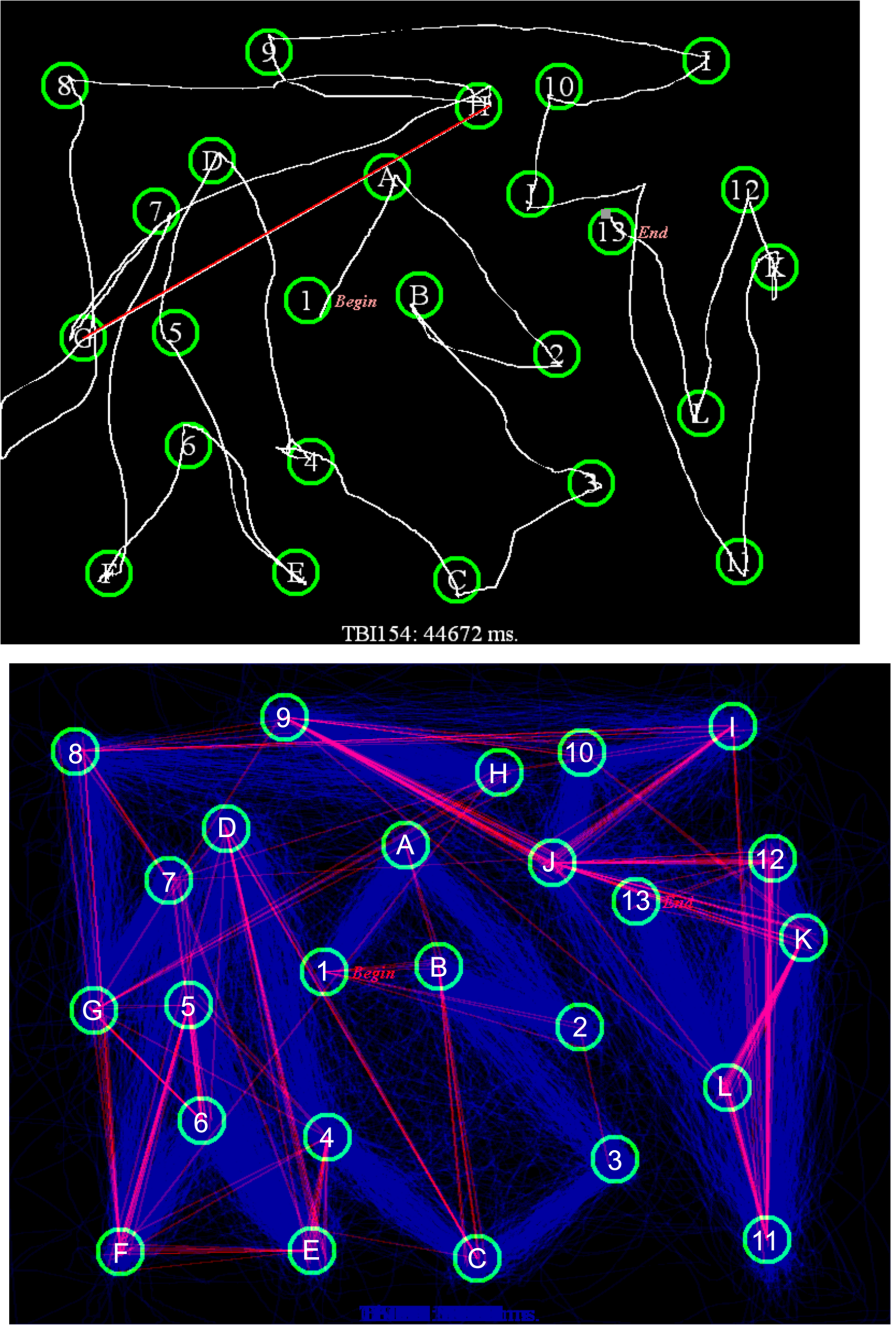
C-TMT-B paths. **A.** A C-TMT-B path from a control subject containing an error shown in red. The subject drew a direct path from G to H (circling through 7) and was then returned to G (straight white line). No further errors were committed. **B.** The superimposed C-TMT-B paths of Experiment 1 control subjects. Paths drawn by individual subjects are shown in blue. Erroneous connections are shown as straight red lines.

Fig 3A shows the C-TMT-B path drawn by a single control subject, and Fig 3B shows the paths from all subjects in Experiment 1. S1 Table (bottom) shows the average metrics for the separate segments of the C-TMT-B, while Table 3 (bottom) shows the correlation matrix for performance on the different segments. As with the C-TMT-A, longer segments had longer completion times [r = 0.68, t(22) = 4.35, p < 0.0003], due primarily to increased move-time [r = 0.76, t(22) = 5.49, p < 0.0001]. Velocity increased with path length [r = 0.72, t(22) = 4.87, p < 0.0001], and was negatively correlated with circuitousness [r = -0.51, t(22) = 2.78, p < 0.01]. Unlike the C-TMT-A, completion times over successive segments did not decrease throughout the test [r = 0.03, NS], nor were there significant changes in dwell-time, move-time, line circuitousness, or velocity.

#### Trails A: analysis by subject

C-TMT-A completion times and other metrics are shown in Table 4 (top). Completion times in Experiment 1 averaged 37.4 s, divided into dwell-time (25.5%), move-time (68.5%), and error time (6.0%).

**Table 4:** Average values from all experiments.

**Trails A**												
	**Type**	**Time**	**SD**	**Dwell**	**Move**	**E-Time**	**Circuit**	**Vel**	**Time-Z**	**Vel-Z**	**Abn-T**	**Abn-V**
**Exp 1.**	Cont	37.39	15.52	9.54	25.60	2.24	1.35	0.30	0.00	0.00	5.4%	5.4%
**Exp. 2a**	Cont	30.28	7.11	8.57	20.54	1.16	1.29	0.34	-0.08	-0.17	4.0%	4.0%
**Exp. 2b**	Cont	26.55	4.60	7.76	18.41	0.38	1.21	0.34	-0.56	-0.28	2.0%	8.0%
**Exp. 2c**	Cont	26.63	6.20	7.85	18.05	0.72	1.22	0.35	-0.60	-0.55	0.0%	6.0%
**Exp. 3**	Mal	47.81	16.76	16.52	28.11	3.18	1.26	0.25	1.65	1.69	44.0%	48.0%
**Exp. 4**	TBI	36.42	9.74	9.40	24.99	2.03	1.28	0.29	0.18	0.44	7.1%	17.9%
**Trails B**												
**Exp 1.**	Cont	60.82	29.50	16.42	38.78	5.58	1.46	0.24	0.00	0.00	5.4%	5.4%
**Exp. 2a**	Cont	46.75	16.17	14.55	29.46	2.75	1.35	0.28	-0.20	-0.18	6.0%	8.0%
**Exp. 2b**	Cont	38.74	8.95	12.15	24.99	1.60	1.24	0.29	-0.71	-0.60	0.0%	6.0%
**Exp. 2c**	Cont	37.42	10.21	11.63	23.73	2.07	1.24	0.31	-0.85	-1.04	0.0%	0.0%
**Exp. 3**	Mal	66.45	30.44	20.53	36.22	9.70	1.28	0.23	0.78	0.84	12.0%	30.0%
**Exp. 4**	TBI	68.23	36.25	18.12	40.39	9.72	1.33	0.23	0.41	0.55	14.3%	21.4%
**Trails B–Trails A**												
**Exp 1.**	Cont	23.42	25.30	6.88	13.18	3.36	0.11	-0.06	0.00	0.00	5.4%	5.4%
**Exp. 2a**	Cont	16.47	15.20	5.97	8.91	1.59	0.07	-0.06	-0.13	-0.01	6.0%	10.0%
**Exp. 2b**	Cont	12.19	8.44	4.39	6.58	1.22	0.03	-0.05	-0.15	-0.31	2.0%	0.0%
**Exp. 2c**	Cont	10.79	8.70	3.77	5.67	1.34	0.03	-0.04	-0.26	-0.49	0.0%	0.0%
**Exp. 3**	Mal	18.64	20.37	4.01	8.11	6.52	0.02	-0.02	-0.87	-0.85	0.0%	0.0%
**Exp. 4**	TBI	31.81	30.16	8.72	15.40	7.69	0.05	-0.06	0.24	0.11	10.7%	0.0%

Type: Cont = control. Mal = malingering. TBI = patients with traumatic brain injury. Time = total completion time. SD = standard deviation. E-Time = error time. Circuit = circuitousness (ratio of length of lines draw to total distance between circles). Vel = velocity. Time-Z = completion-time adjusted for age and computer use. Vel–z = velocity z-score adjusted for age and computer use. Abn-T = percentage of subjects with abnormal (p < 0.05) completion time z-scores. Abn-V = percentage of subjects with abnormal (p < 0.05) velocity z-scores.

**Fig 4:**
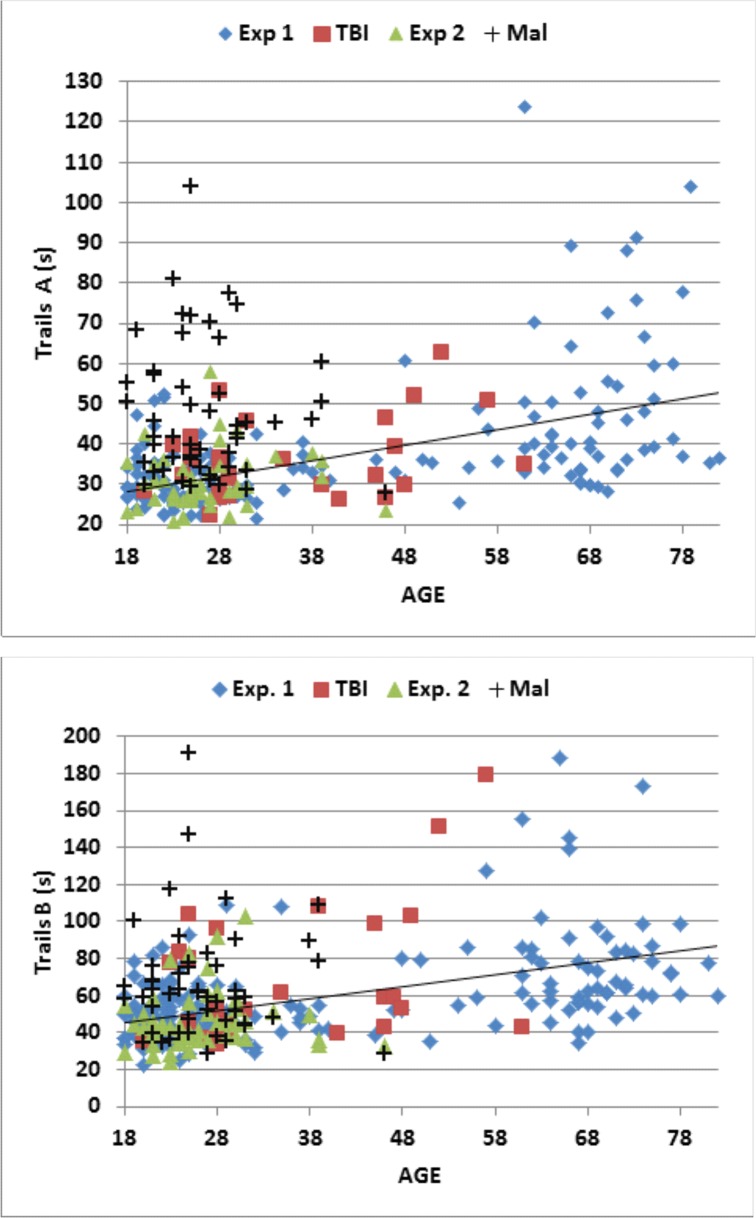
C-TMT completion times for Trails A (top) and Trails B (bottom). Shown as a function of age. The data are from Experiment 1 (blue diamonds), the first session of Experiment 2 (green triangles), malingering subjects in Experiment 3 (Mal, black crosses), and TBI patients in Experiment 4 (red squares). The linear age-regression functions from Experiment 1 are shown.

Fig 4 (top, blue diamonds) shows the increase in C-TMT-A completion times as a function of age [0.28 s/year, r = 0.53, t(163) = 7.98, p < 0.0001]. The completion times of the oldest subjects were increased by 18.5 s with respect to the youngest subjects, a difference slightly more than one standard deviation in the population as a whole.

Table 5 (top) shows the correlation matrix for factors influencing C-TMT-A completion time: age had a significantly larger effect than any other demographic factor, including computer-use [r = 0.53 vs. r = -0.35, z = 2.02, p < 0.05]. Further analysis showed that age-related increases were due more to changes in move-time than dwell-time [r = 0.58 vs. r = 0.30, z(163) = 3.19, p < 0.002], with large age-related reductions in velocity [r = -0.64, t(163) = -10.63, p < 0.0001]. Older subjects were less impaired at detecting targets than in drawing connecting lines, resulting in an age-related decrease in the dwell-time/total-time ratio [r = -0.22, t(163) = -2.89, p < 0.005]. There was also a small age-related increase in circuitousness [r = 0.28, t(163) = 3.74, p < 0.0003].

**Table 5:** Correlation matrix of CTMT-A and C-TMT-B metrics from Experiment 1.

**TRAILS A**									
	**Edu**	**C-use**	**Time**	**Dwell**	**Move**	**Circuit**	**Vel**	**E-Time**	**Dwell/tot**
**Age**	0.37	-0.20	0.53	0.30	0.58	0.28	-0.64	0.21	-0.22
**Edu**		0.17	0.18	0.09	0.11	0.09	-0.14	0.18	0.03
**C-Use**			-0.35	-0.23	-0.43	-0.27	0.39	-0.05	0.17
**Time**				0.57	0.86	0.55	-0.67	0.69	-0.13
**Dwell**					0.40	0.05	-0.52	0.17	0.63
**Move**						0.65	-0.74	0.30	-0.40
**Circuit**							-0.13	0.27	-0.43
**Vel**								-0.17	0.15
**E-Time**									-0.07
**TRAILS B**									
	**Edu**	**C-use**	**Time**	**Dwell**	**Move**	**Circuit**	**Vel**	**E-Time**	**Dwell/tot**
**Age**	0.37	-0.20	0.48	0.22	0.54	0.33	-0.62	0.22	-0.17
**Edu**		0.17	0.06	0.10	0.06	0.02	-0.13	0.00	0.09
**C-Use**			-0.28	-0.15	-0.36	-0.14	0.41	-0.08	0.12
**Time**				0.43	0.92	0.54	-0.59	0.82	-0.22
**Dwell**					0.21	-0.11	-0.39	0.10	0.70
**Move**						0.68	-0.65	0.64	-0.48
**Circuit**							-0.12	0.41	-0.53
**Vel**								-0.24	0.12
**E-Time**									-0.29

Edu = education. C-use = computer-use. Time = total completion time. Vel = velocity. E-time = error time. Dwell/tot = ratio of dwell time to total time. Given the sample size (N = 165), correlations with |r| > 0.16 are statistically significant at the p < 0.05 level (uncorrected). See Table 4 for an explanation of other abbreviations.

Completion times were significantly reduced with increased computer-use [r = -0.43, t(163) = 6.08, p < 0.0001]. Increased computer-use reduced move-times [r = -0.43, t(163) = 6.08, p < 0.0001] more than dwell-times [r = -0.27, t(163) = 3.58, p < 0.0005], which increased the dwell-time/total-time ratio [r = 0.18, t(163) = 2.21, p < 0.05]. Reduced move-times were due to increased drawing velocities [r = 0.39, t(163) = 5.41, p < 0.0001], as well as reduced circuitousness [r = -0.27, t(163) = -3,59, p < 0.0005]. The largest differences were between the 3.6% of subjects who used computers “less than 1 hour per week”, and the 5.6% of subjects who used computers “less than 1 hour per day”. Only small differences were seen between the remaining subjects, who used computers regularly (i.e., between 1 hour/day and 8 hours/day). Sex had no significant influences on completion times [r = -0.11], and education had insignificant effects when considered conjointly with age and computer-use [t(161) = 0.76], with both age and computer-use remaining significant [t(161) = 6.48, and t(161) = -3.94, p < 0.0002 for both comparisons].

Errors on C-TMT-A were infrequent (mean 0.63/test), and occurred in 34.7% of subjects. Path analysis showed that most errors were “near misses”; i.e., subjects failed to fully connect a circle (e.g., Fig 2). On average, each error added 3.56 s to completion time. Errors increased in subjects with slower completion times (excluding errors) [r = 0.27, t(163) = 3.59, p < 0.0005] and in subjects who drew more circuitously [r = 0.33, t(163) = 4.48, p < 0.0001], but error incidence did not correlate with movement velocity [r = -0.10, NS]. Errors increased slightly with age [r = 0.21, t(163) = 2.75, p < 0.01], but the incidence of errors was not significantly influenced by education, sex, or computer-use [|r| < 0.13 for all comparisons].

**Fig 5:**
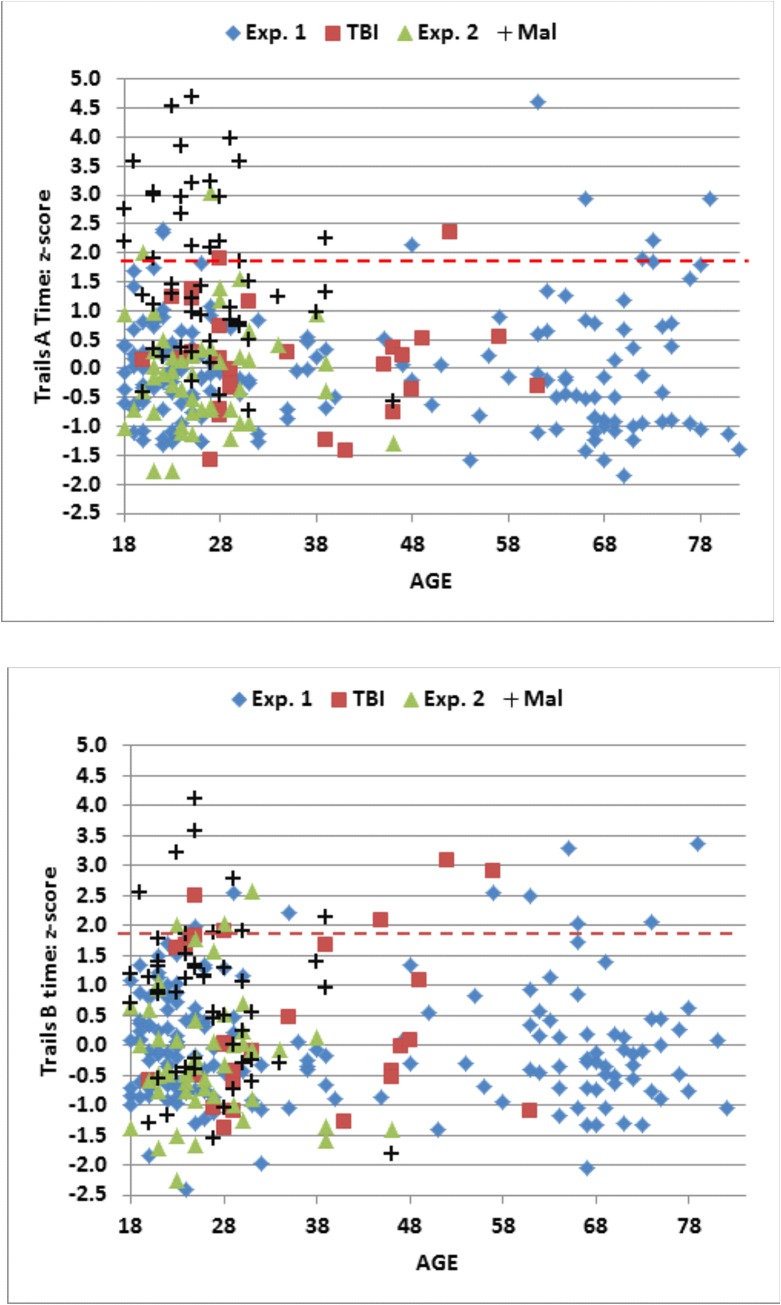
C-TMT completion time z-scores for Trails A (top) and Trails B (bottom). Shown as a function of age. Regression functions from Experiment 1 were used to adjust Z-scores for age and computer use. Dashed lines show p< 0.05 abnormality thresholds.

Because completion times were positively skewed (skew = 2.60), they were log transformed before further statistical analysis. Log-transformed completion times (skew = 1.17) showed strong correlations with age [r = 0.59, t(163) = 9.36, p < 0.0001] and computer-use [r = -0.39, t(163) = -5.42, p < 0.0001], with the correlation with age stronger than that with computer-use [z = 2.4, p < 0.02]. Linear regression analysis showed that age and computer-use conjointly accounted for 42.5% of the C-TMT-A log-completion time variance, with both making significant individual contributions [Age, t(163) = 8.78, p < 0.0001; Computer-use, t(163) = -4.70, p < 0.0001]. Individual z-scores, calculated from age- and computer-use regressed log completion times, are shown for subjects of different ages in Fig 5 (top, blue diamonds). Z-scores did not correlate significantly with sex, education, or handedness. As expected, z-scores correlated with dwell-time [r = 0.54, t(163) = 8.22, p < 0.0001] and move-time [r = 0.58, t(163) = 9.22, p < 0.0001], reflecting proportional increases in both measures: i.e., there was no significant correlation between z-scores and the dwell-time/total-time ratio [r = 0.09, NS].

**Fig 6:**
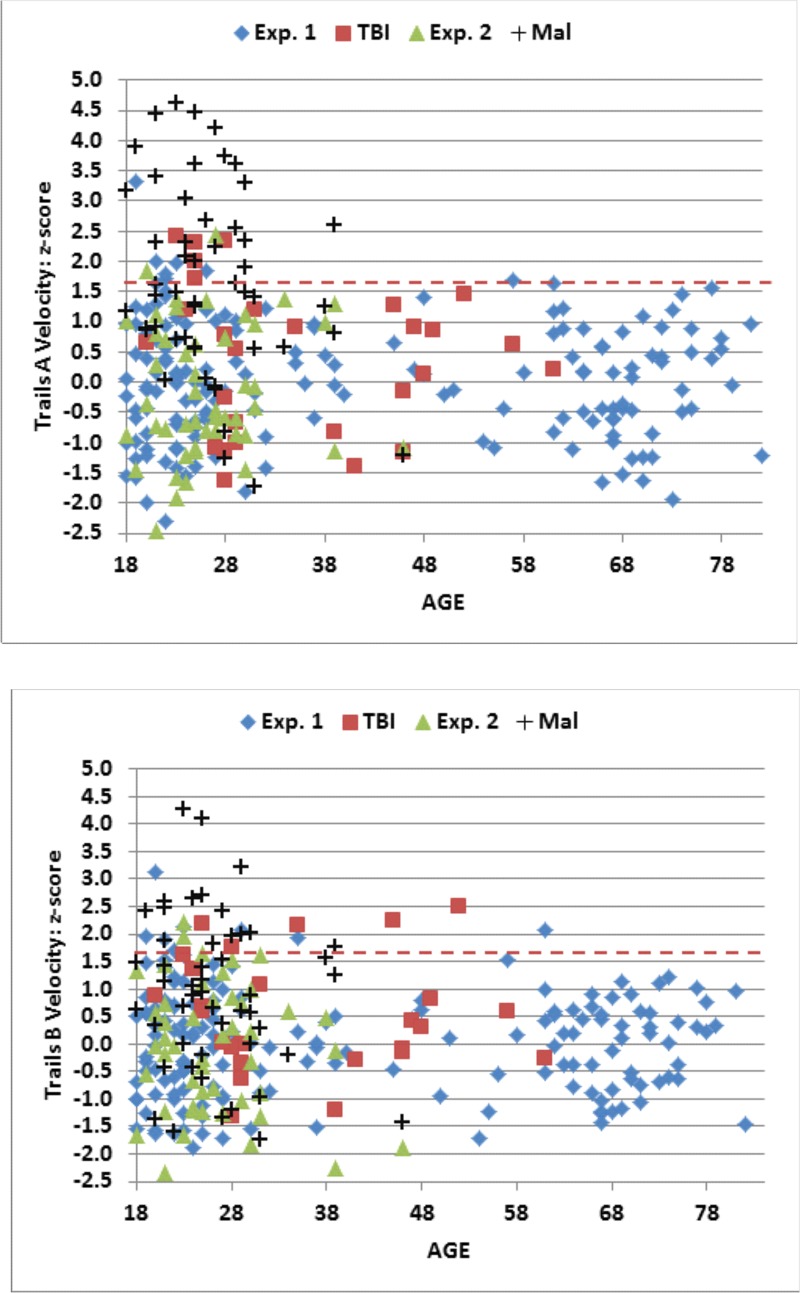
C-TMT velocity z-scores for Trails A (top) and Trails B (bottom). Shown as a function of age. Regression functions from Experiment 1 were used to adjust Z-scores for age and computer use. Dashed lines show p< 0.05 abnormality thresholds.

Mean C-TMT-A velocity (skew = 0.0) also correlated strongly with age [r = -0.64, t(163) = -10.67, p < 0.0001], and to a lesser degree with computer-use [r = 0.39, t(163) = 5.42, p < 0.0001]. Multiple regression analysis showed that age and computer-use accounted for 47.9% of velocity variance, with both factors making significant individual contributions [age, t(162) = -10.12, p < 0.0001; computer-use, t(162) = -4.73, p < 0.0001]. Velocity z-scores were calculated from age- and computer-use regressed velocity values, and are shown for subjects of different ages in Fig 6 (top, blue diamonds). Velocity z-scores did not vary significantly with education or handedness, but were slightly increased in men [r = 0.16, t(163) = 2.08, p < 0.05]. There was no significant correlation of velocity z-scores with the dwell-time/total-time ratio [r = 0.04, NS].

#### C-TMT-B: analysis by subject

C-TMT-B completion times are shown in Table 4 (center). Completion times averaged 60.8 s, divided into dwell-time (27.0%), move-time (63.8%), and error time (9.2%).

Table 5 (bottom) shows the correlation matrix for factors influencing C-TMT-B completion times, and Fig 4 (bottom, blue diamonds) shows C-TMT-B completion times as a function of age. As with the C-TMT-A, completion times increased markedly with age [0.48 s/year, r = 0.48, t(163) = 7.01, p < 0.0001]. Thus, the completion times of the oldest subjects were increased by slightly more than one standard deviation with respect to the youngest subjects. As with the C-TMT-A, age-related increases were larger for move-time than dwell-time [r = 0.54 vs. r = 0.22, z = 3.44, p < 0.0006], so that increasing age reduced the dwell-time/total-time ratio [r = -0.17, t(163) = 2.21, p < 0.03]. Older subjects also showed increased circuitousness [r = 0.33, t(163) = 4.48, p < 0.0001].

Completion times on the C-TMT-B were significantly reduced with increased computer-use [r = -0.28, t(163) = 3.74, p < 0.0003], without a significant influence of computer-use on the dwell-time/total-time ratio [r = 0.12, t(163) = 1.55, p < 0.15], or, unlike the C-TMT-A, on line circuitousness [r = -0.14, t(163) = -1.81, p < 0.10]. Again, the largest effects of computer-use were seen in subjects who rarely or never used computers. Neither education nor handedness had significant influences on C-TMT-B completion times [|r| < 0.14 t(163) = -1.81, p < 0.10 for both comparisons], but completion times were slightly reduced in male subjects [r = -0.18, t(163) = 2.34, p < 0.03].

Errors were increased with respect to their incidence on the C-TMT-A [mean 1.31/test, p < 0.0003], with 54.2% of subjects committing at least one error. On average, each error added 4.25 s to completion time. While a significant proportion of errors reflected clicks outside circles, perseverative errors (i.e., subjects failing to alternate between letters and numbers [29]) were also evident (Fig 3A). Errors were increased in subjects with slower completion time (excluding error time [r = 0.46, t(163) = 6.63, p < 0.0001]), and were slightly reduced in subjects with greater education [r = -0.16, t(163) = 2.08, p < 0.05]. Error time increased somewhat with age [r = -0.17, t(163) = 2.20, p < 0.02], but was not significantly influenced by computer-use [r = -0.08, NS].

Because completion times were positively skewed (skew = 2.73), they were log transformed to reduce skew and kurtosis before further analysis. Log-transformed completion times (skew = 0.76) continued to show a strong relationship to age [r = 0.57, t(163) = 8.88, p < 0.0001] and computer-use [r = -0.37, t(163) = 5.10, p < 0.0001], with age effects again showing a stronger influence than computer-use [z = 2.34, p < 0.02]. Multiple regression analysis showed that the two factors accounted for 32.3% of log-completion time variance, with both making significant individual contributions [Age, t(163) = 7.24, p < 0.0001; Computer-use, t(163) = -3.50, p < 0.0007]. Individual z-scores were calculated from age- and computer-use regressed completion times, and are shown for subjects of different ages in Fig 5 (bottom, blue diamonds). Z-scores did not correlate significantly with sex, education, handedness, or the dwell-time/total-time ratio.

Mean C-TMT-B velocity (skew = 0.08) also correlated with age [r = -0.62, t(163) = -10.12, p < 0.0001] and computer-use [r = 0.41, t(163) = 5.72, p < 0.0001]. Multiple regression analysis showed that age and computer-use accounted for 46.3% of velocity variance, with both factors making significant individual contributions [Age, t(162) = -9.51, p < 0.0001; Computer-use, t(162) = -5.06, p < 0.0001]. Velocity z-scores, calculated from age- and computer-use regressed velocity values, are shown in Fig 6 (bottom, blue diamonds). Velocity z-scores did not vary significantly with education, handedness, or dwell-time/total-time ratios. However, as with the C-TMT-A, men moved slightly faster than women [r = 0.16, t(163) = 2.08, p < 0.05].

#### C-TMT-A vs. C-TMT-B

C-TMT-A and C-TMT-B completion times were strongly correlated [r = 0.55, t(163) = 8.43, p < 0.0001], as were dwell-times [r = 0.37, t(163) = 5.10, p < 0.0001], move-times [r = 0.67, t(163) = 11.56, p < 0.0001], the dwell-time/total-time ratio [r = 0.42, t(163) = 5.93, p < 0.0001], and particularly movement velocities [r = 0.84, t(163) = 19.83, p < 0.0001]. However, neither the number of errors [r = 0.02] nor error time [r = 0.08] showed significant inter-test correlations.

Comparisons of performance on the C-TMT-B and C-TMT-A revealed the expected increases in the number of C-TMT-B errors [F(1,164) = 14.90, p < 0.0002, ω^2^ = 0.08] and error time [F(1,164) = 10.12, p < 0.002, ω^2^ = 0.05], but also increases in line circuitousness [F(1,164) = 16.02, p < 0.0001, ω^2^ = 0.08] and the dwell-time/total-time ratio [F(1,164) = 16.43, p < 0.0001, ω^2^ = 0.09].

Table 4 (bottom) shows summary statistics for the C-TMT-B minus C-TMT-A difference (mean = 23.4 s). Difference times increased with age [r = 0.23, t(163) = 3.03, p < 0.003], were slightly shorter in men [r = -0.16, t(163) = 2.08, p < 0.04], and were not significantly affected by education or computer-use. The C-TMT-B/C-TMT-A ratio averaged 1.69 and, as in previous reports [18], was not affected by age, nor was it significantly affected by education, sex, or computer-use.

### Discussion: Experiment 1

#### Overview

C-TMT-A completion times (37.4 s) were somewhat longer than those of traditional TMTs (see Table 1). Three factors were likely responsible: (1) Subjects drew connecting lines with the mouse rather than with the more familiar pencil; (2) Errors were scored more stringently and occurred with somewhat greater frequency on the C-TMT-A than on the manually administered Trails A; and (3) Subjects were required to stop and depress the mouse button inside each circle.

In contrast to the C-TMT-A, C-TMT-B completion times (60.8 s) were reduced in comparison with completion times on traditional TMTs (see Table 1). Four factors were likely responsible: (1) The total length of the C-TMT-B path was equated to that of the C-TMT-A path, whereas in the standard TMT, the path length of Trails B exceeds that of Trails A (see above); (2) The search for unconnected circles was facilitated by color cues: i.e., circles that had already been selected were colored green, whereas unselected circles were colored white; (3) Errors were corrected automatically and more rapidly than in manual testing; and (4) Display clutter was reduced because the connecting lines drawn by subjects were replaced by straight green lines, and lines drawn in error were erased.

#### Path analysis of the C-TMT-A and C-TMT-B

Path analysis revealed a number of performance variations on individual test segments that related to path length. Not surprisingly, longer segments took longer to connect, due primarily to increases in move-time. However, movement velocity increased with path length, and longer lines were less circuitous. Moreover, on the C-TMT-A, the dwell-time/total-time ratio was reduced on longer paths. This indicates that the path length of TMT segments influenced the relative time spent finding target circles and connecting them.

In contrast to the traditional Trails A [7], C-TMT-A subjects increased their speed on successive segments, due primarily to reductions in dwell-time. This suggests that the detection of target circles was facilitated as the test progressed because potential targets were distinguished by color (unconnected circles were white while previously connected circles were green), and by the absence of connecting lines. In contrast, no acceleration was observed on later segments of the C-TMT-B. This may be because subjects were more familiar with number/letter relations occurring early in the test (e.g., 1-A, 2-B) than those occurring later (e.g. I-10, J-11) [7]. As a result, the effects of increased circle salience due to the color cues may have been counteracted by greater difficulty in number/letter sequencing.

#### Errors

We found increased error rates on the C-TMT-A and C-TMT-B (0.63 and 1.31 per test, respectively) in comparison with traditional TMTs [26, 28, 29], which likely reflected more stringent scoring: the majority of errors in the C-TMT were due to subjects clicking outside a circle’s circumference. Such errors are not always scored on the manually administered TMT.

#### The effects of aging

As in previous studies (e.g., see Table 1), completion times increased markedly as a function of age on both the C-TMT-A and C-TMT-B. The correlations that we obtained between age and completion time (C-TMT-A = 0.53, C-TMT-B = 0.48) were very similar to the correlations reported in previous studies [4, 21]. We found that the primary effect of aging was to slow move-times. Move-times showed a larger relative increase in older subjects than did dwell-times, resulting in significant age-related reductions in the dwell-time/total-time ratios on both the C-TMT-A and C-TMT-B. Further analysis showed that age-related increases in move-times primarily reflected decreases in movement velocity, accompanied by smaller increases in line circuitousness. Older subjects also showed slight increases in error rates. As in previous studies [4], aging also resulted in increases in the C-TMT-B minus C-TMT-A difference, reflecting the steeper slope of age-related changes in C-TMT-B completion times.

#### The effects of computer-use

Computer-use also had significant effects on C-TMT performance. This presumably reflects increased familiarity with using the mouse to access elements in computer displays. Increased computer use primarily reduced move-times, resulting in an increase in the dwell-time/total-time ratios on both the C-TMT-A and C-TMT-B. Move-time reductions were due primarily to increases in movement velocity, accompanied by smaller reductions in line circuitousness.

#### The effects of education

In contrast to some studies [4, 11, 21, 31, 32] but in accord with others [33], we found that education had minimal influence on completion times for either the C-TMT-A or C-TMT-B. In previous studies, education effects are much smaller than the effects of age [4], and are most pronounced for subjects with very limited (e.g., grade school) education [10, 21, 22, 25, 31, 34]. Our subjects had very high overall levels of education, with more than 98% having completed high school and more than 50% having attended at least two years of college. Moreover, most of the younger subjects were still in college. As a result, older subjects had greater education and coincidentally tended to use computers less (see Table 5). Since both age and computer-use had strong influences on completion times, their combined influence was stronger than the effects of education.

## Experiment 2: Generalization and Test/Retest Reliability

In clinical applications, the results of a patient’s manually administered TMT are compared with the normative results from demographically similar control subjects [32]. However, the substantial differences in TMT norms gathered at different sites (see Table 1) suggest that differences in test administration by individual examiners may significantly influence results. For example, the results of Ising, Mather [6] show significant differences in TMT completion times in two demographically similar German control populations tested by different examiners. In contrast, C-TMT administration is fully automated. As a result, no significant differences would be expected when C-TMT test results are compared in different groups of demographically similar control subjects. Therefore, one goal of Experiment 2 was to determine if the results of Experiment 1 would fit the results of an independent group of 50 younger and slightly better educated control subjects.

A second requirement for the clinical application of a neuropsychological test is the reliability of test results, which is evaluated with test-retest reliability estimates. Previous studies have found high test-retest reliability for the standard TMT when administered in the same session [21], at intervals of one day [22], and at intervals of one month [25]. To evaluate the test-retest reliability of the C-TMT, the 50 control subjects of Experiment 2 underwent two additional test sessions. This also permitted the examination of learning effects on C-TMT performance, as previous studies have established that standard TMT performance improves significantly with repeated testing [35].

### Methods: Experiment 2

#### Subjects

Fifty young volunteers (mean 26.3 years, range 18–46 years, 52% male) were recruited primarily from internet advertising, using exclusion criteria identical to those of Experiment 1. They volunteered to participate in three test sessions at weekly intervals to evaluate test-retest reliability, and in a fourth session to study the effects of malingering (see Experiment 3, below). Their demographic characteristics are shown in Table 2. The majority of subjects were young college students, with slightly more education and significantly greater levels of computer-use [p< 0.03] than the subjects in Experiment 1. Ethnically, 68% were Caucasian, 11% Latino, 9% African American, 10% Asian, and 2% other. Other methods were identical to those of Experiment 1.

### Results: Experiment 2

#### Comparisons with normative data from Experiment 1

**Fig 7:**
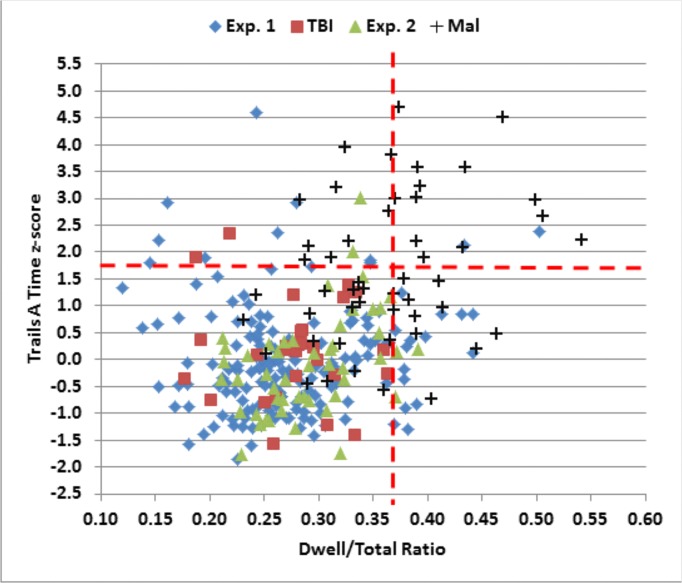
C-TMT-A completion-time z-scores as a function of the dwell-time/total time ratios. The dashed horizontal line shows the p < 0.05 abnormality threshold for completion time. The dashed vertical line shows the p < 0.10 threshold for the dwell-time/total-time ratio.

**Fig 8:**
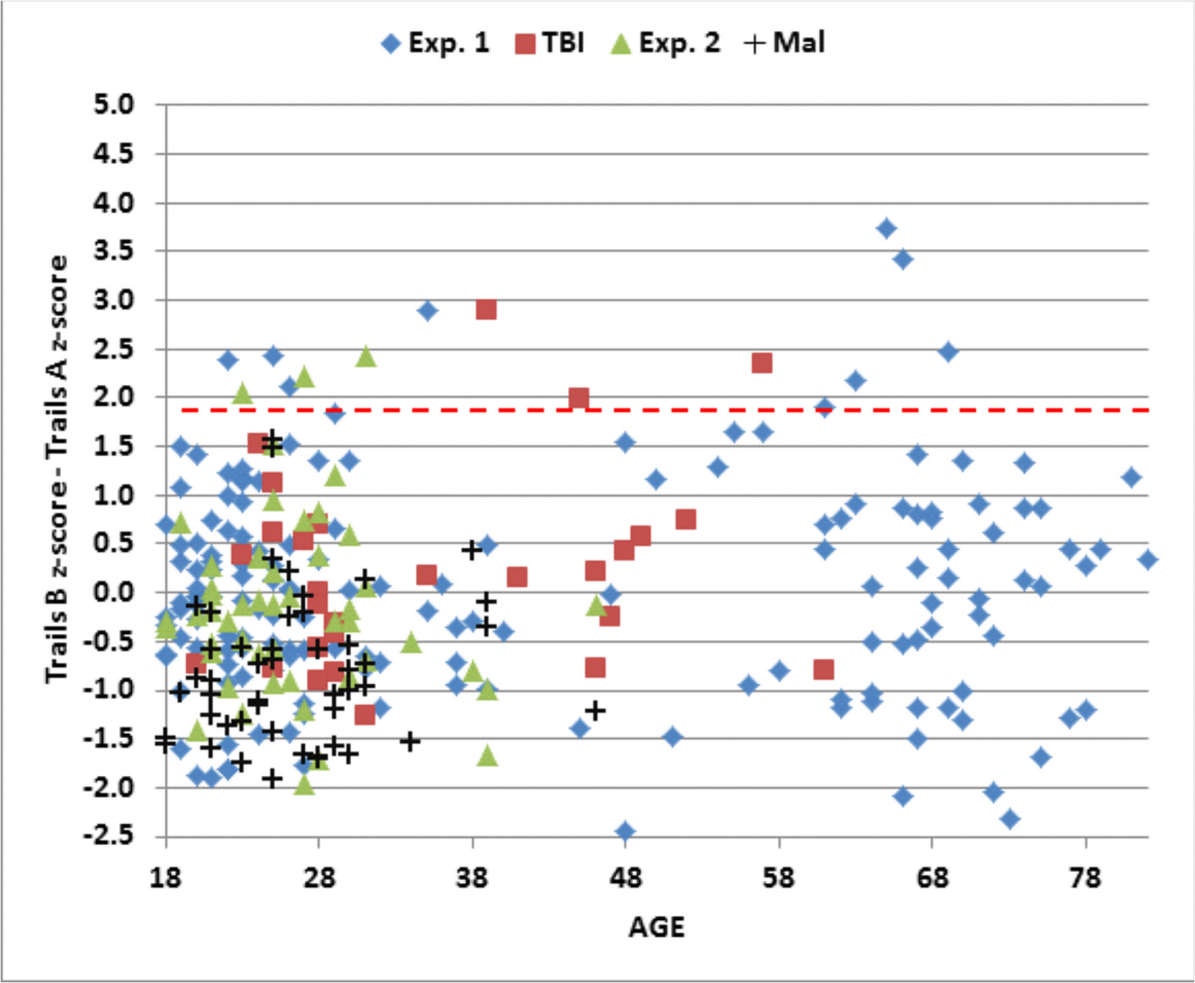
Difference z-scores (C-TMT-B minus C-TMT-A) for subjects of different ages. The dashed horizontal line shows the p< 0.05 abnormality threshold.

The data from individual subjects in Experiment 2a (green triangles) are included in Figs 4–6, and summary statistics are included in Table 4. Fig 7 shows completion-time z-scores as a function of the dwell-time/total-time ratio, and Fig 8 shows differences in C-TMT-B and C-TMT-A z-scores as a function of age.

Age- and computer-use adjusted z-scores did not differ significantly from those obtained in Experiment 1 on either the C-TMT-A or the C-TMT-B. However, the increased homogeneity of age, education, and computer-use of Experiment 2 subjects was associated with a substantial reduction in completion-time standard deviations, as shown in Table 4 (45.8% and 54.8% of the standard deviations seen in Experiment 1 for the C-TMT-A and C-TMT-B, respectively). There were no significant differences from Experiment 1 in error time or dwell-time/total-time ratios. The percentage of Experiment 2 subjects showing abnormal performance at p< 0.05 cutoffs ranged from 4% to 8% on z-score and velocity z-score measures (Table 4). In short, the data from Experiment 2a was well-fit by the regression functions obtained in Experiment 1.

#### Test-retest reliability

C-TMT-A metrics showed high reliability across test sessions. For total-time z-scores, the intraclass correlation coefficient (ICC) was 0.87. High reliability was also seen for velocity z-scores (ICC = 0.84). Other metrics also showed moderate to high reliability, including dwell-time (ICC = 0.79), move-time (ICC = 0.74), the dwell-time/total-time ratio (ICC = 0.86), and, to a lesser degree, circuitousness (ICC = 0.62). In contrast, error-times were unreliable (ICC = -0.17). Intrasubject variance was estimated from the standard deviation of the results of the three tests for each subject. The average within-subject standard deviation in completion times for the C-TMT-A was 2.67s.

C-TMT-B metrics showed similarly high test-retest reliability for total-time z-scores (ICC = 0.85) and velocity z-scores (ICC = 0.90). Other metrics also showed significant test-retest correlations, including dwell-time (ICC = 0.76), move-time (ICC = 0.75), and dwell-time/total-time ratios (ICC = 0.74). However, lower reliability was seen for circuitousness (ICC = 0.65), and particularly for error time (ICC = 0.33). Intraclass correlation coefficients for difference measures between the C-TMT-B and C-TMT-A were reduced, e.g., for total-time z-scores (ICC = 0.56) and velocity (ICC = 0.48). The average within-subject standard deviation in completion times for the C-TMT-B was 5.34 s.

#### Learning effects

Learning effects on the different C-TMT metrics are summarized in Table 6. Performance improved on successive test sessions (Experiment 2b and 2c), as shown in Table 4. On the C-TMT-A, total time was reduced by 12% (p < 0.001) between Experiments 2a and 2b, due to reduced dwell- and move-times, reduced circuitousness, and increased velocity. No significant differences in completion time were seen between Experiments 2b and 2c. Mean z-score improvements amounted to -0.52 for completion time and -0.38 for velocity when Experiment 2a and Experiment 2c were compared. Performance improvements were somewhat larger on the C-TMT-B, reducing completion times by 20% overall, with performance improvements again concentrated between Experiments 2a and 2b, and overall z-score changes of -0.68 for completion time and -0.82 for velocity. Neither education nor computer-use were significantly correlated with the improvements between sessions 2a and 2b on either the C-TMT-A or C-TMT-B (r< |0.2| for all comparisons).

**Table 6:** Changes in performance across the repeated test sessions in Experiment 2.

**Trails A**					
	**Time**	**Dwell**	**Move**	**Circuit**	**Vel**
**Exp. 2a**	30.28	8.57	20.54	1.29	0.34
**Exp. 2b**	88%	90%	90%	94%	102%
**Exp. 2c**	88%	92%	88%	95%	105%
**Trails B**					
**Exp. 2a**	46.75	14.55	29.46	1.35	0.28
**Exp. 2b**	83%	84%	85%	92%	106%
**Exp. 2c**	80%	80%	81%	92%	113%

Mean values are shown for Experiment 2a, and percentages of values relative to the Experiment 2a test session are shown for repeated tests (2b and 2c).

### Discussion: Experiment 2

After factoring out the influences of age and computer-use with the regression functions obtained in Experiment 1, the z-scores of the control subjects in Experiment 2a did not differ significantly from Experiment 1 subjects for any of the C-TMT-A or C-TMT-B metrics, including completion time and velocity. The incidence of abnormal scores (4% to 8%) was also within the expected (p< 0.05) range. This suggests that the normative data from Experiment 1, when corrected for age and computer-use, provided a good fit to the data from a different population of younger and more computer-experienced control subjects in Experiment 2.

Intraclass correlations for total-time and velocity z-scores were somewhat higher than those reported in previous manually administered TMT studies [21, 22, 25]. In addition, other metrics, including dwell-time, move-time, and dwell-time/total-time ratios, showed good replicability. However, errors occurred infrequently and error time did not correlate significantly across test sessions.

As in previous studies [36], we found significant learning effects on repeated TMT administration, with most learning occurring over the first two test sessions and greater learning on the C-TMT-B than on the C-TMT-A. Although alternate C-TMT test versions might diminish these learning effects to some extent, recent studies have found evidence that learning even occurs across alternative test forms [15]. Hence, learning effects must be taken into consideration when interpreting the results of repeated C-TMT test administration.

## Experiment 3: Effects of Malingering

Once a patient’s neuropsychological test results show that performance falls into the abnormal range, the examiner is faced with the challenge of determining whether the poor performance is due to impaired ability or suboptimal effort. The detection of malingering is particularly important in the evaluation of patients with head injury, where litigation and pension claims can provide substantial financial incentives to malinger [37]. When patients are given a series of neuropsychological tests, malingering effects are more prominent on some tests than others, with a relatively low incidence of malingering seen with the TMT [38]. Subjects who malinger show prolonged TMT completion times [38], with greater elevations often seen in Trails A than in Trails B [39–41]. The number of errors also increases in malingering subjects [28, 42].

One approach to malingering detection is to give subjects additional symptom validity tests, such as the Test of Memory Malingering (TOMM) [43]. However, in recent years, information about symptom validity tests has become widely available on the internet, so that motivated subjects can learn to avoid detection. In the current experiment, we evaluated whether additional C-TMT metrics, independent of overall completion time, could assist in distinguishing malingering subjects from controls. For example, malingering subjects could slow completion times by resting on successive circles (i.e., increasing dwell-time), drawing slowly (reducing movement velocity), drawing circuitously, or simply by making more errors. Observable differences in the causes of slowed completion time in malingering subjects and control subjects with abnormal completion times would assist in determining if slowed performance was due to malingering or organic causes.

### Methods: Experiment 3

#### Subjects

Subjects from Experiment 2 participated one week later in Experiment 3, after receiving instructions to perform like a patient with a minor head injury. Their instructions were as follows: “Listed below you’ll find some of the symptoms common after minor head injuries. Please study the list below and develop a plan to fake some of the impairments typical of head injury when you take the next test. Do your best to make your deficit look realistic. If you make too many obvious mistakes, we’ll know you’re faking! Symptom list: Difficulty concentrating for long periods of time, easily distracted by unimportant things, headaches and fatigue (feeling “mentally exhausted”), trouble coming up with the right word, poor memory, difficulty performing complicated tasks, easily tired, repeating things several times without realizing it, slow reaction times, trouble focusing on two things at once.” Malingering subjects took the entire battery of cognitive tests following the procedures described in Experiment 1.

### Results: Experiment 3

#### C-TMT-A

Table 4 includes the results from Experiment 3. Data from the malingering subjects (black crosses) are shown in the scatter plots in Figs 4–8. Malingering subjects showed a large increase in mean C-TMT-A completion times (mean z-score = +1.65) and reductions in velocity (z-score = -1.69). In comparison with the baseline results from the same subjects (Experiment 2a), overall completion time increased by 58%, dwell-time increased by 136%, and move-time increased by 37%. Errors (mean 1.54) also increased, accompanied by a 174% increase in error-time.

Overall, 44% (22) of malingering subjects produced abnormal completion-time z-scores (p < 0.05) on the C-TMT-A (Fig 5, top), and a similar percentage produced abnormal C-TMT-A velocities (Fig 6, top). We also examined the dwell-time/total-time ratio, which was uncorrelated with overall completion time in control subjects. As shown in Fig 7, among the 22 malingering subjects with abnormal completion times, the dwell-time/total-time ratio was increased to suggestively abnormal levels (p< 0.10) in 50%. This suggests that many malingering subjects with abnormal completion times paused excessively during the test. In contrast, among the 11 control subjects (4.9%) in Experiments 1 and 2a with abnormal C-TMT-A completion times, nine showed dwell-time/total-time ratios within the normal range. Thus, considering all subjects with abnormal C-TMT-A completion times, the dwell-time/total-time ratio accurately classified 50% of the malingering subjects and 82% of controls.

#### C-TMT-B

As shown in Figs 5 (bottom) and 6 (bottom), malingering subjects showed smaller average deficits on the C-TMT-B than the C-TMT-A, both for total completion time (z-score = 0.78) and velocity (z-score = 0.84). Overall, only nine (18%) malingering subjects produced abnormal C-TMT-B completion times, all of whom also had abnormal C-TMT-A z-scores, including 83% who produced greater z-score abnormalities on the C-TMT-A than the C-TMT-B. In contrast, of the 12 control subjects with abnormal z-scores on the C-TMT-B, only one produced abnormal results on the C-TMT-A, and the abnormalities on the C-TMT-B were uniformly greater than those on the C-TMT-A. Thus, considering only subjects with abnormal scores on the C-TMT-B, the differences of C-TMT-A and C-TMT-B z-scores correctly classified 83% of malingering subjects and 100% of controls.

#### C-TMT-B versus C-TMT-A

Fig 8 shows the differences in z-scores on the C-TMT-B and C-TMT-A. All malingering subjects produced difference z-scores within the normal range. Indeed, because malingering subjects showed greater deficits on the C-TMT-A than on the C-TMT-B, z-score differences were significantly more negative (mean z-score difference = -0.87) than those of controls ([t(112) = -5.79, p < 0.0001], and the C-TMT-B/C-TMT-A ratio was significantly reduced [1.38 vs 1.69 in controls, t(138) = -3.98, p < 0.0001]. Malingerers also showed a greater relative slowing of velocity z-scores on the C-TMT-A than on the C-TMT-B when compared to controls [t(79) = -6.80, p<0.0001].

### Discussion: Experiment 3

Consistent with previous reports [28], subjects who were instructed to malinger produced elevated C-TMT completion times. In the current experiment, 44% of malingering subjects produced abnormal results on the C-TMT-A, and 18% produced abnormalities on the C-TMT-B. As in previous reports [38], malingering-related abnormalities on the TMT were relatively less common than those observed in the same subjects on tests of simple reaction time [44], digit span [45], and finger-tapping [46].

As in previous reports [28, 39, 40, 42, 47], malingering effects were greater on Trails A than Trails B, as reflected in significant reductions in C-TMT-B versus C-TMT-A difference measures, ratios, and movement velocities, a pattern opposite that seen in the majority of patients with brain injury [8]. Indeed, even among malingering subjects with abnormal performance on the C-TMT-B, 89% showed greater z-score abnormalities on the C-TMT-A, whereas none of the control subjects with abnormal C-TMT-B completion times showed greater C-TMT-A abnormalities. This reflected the fact that malingering subjects slowed completion times by similar absolute amounts in the two tests (e.g., 17.5 s on the C-TMT-A and 19.7 s on the C-TMT-B), suggesting that they had difficulty adjusting the magnitude of their “abnormalities” to the difficulty of the task. A similar effect was seen in the same subjects during comparisons of simple and choice-reaction times, where malingering effects were much more significant in the simple reaction time task [48].

How did malingering subjects produce delayed completion times? On the C-TMT-A, 50% of malingering subjects showed abnormally prolonged dwell-time/total-time ratios, a pattern that was rarely seen in controls. This suggests that many malingering subjects paused excessively before beginning to draw connecting paths to the next circle.

## Experiment 4: Effects of Traumatic Brain Injury

The TMT is one of the core measures recommended for use in evaluating cognitive function following traumatic brain injury (TBI) [49], in part because TMT results are predictive of eventual functional outcome [50–52]. TMT completion times show small increases in the acute phase of mild concussion [53] that usually resolve within one week [54]. Mild TBI (mTBI) can prolong TMT completion times in the chronic phase, particularly on Trails B, even in patients with no neuroimaging evidence of brain damage [55]. The increased completion times are not generally accompanied by increases in error frequency [28, 56]. However, prolongations in both Trails A and Trails B can be seen in more complicated mTBI cases [51, 54]. Patients with more severe TBI (sTBI) produce greater slowing, increased error rates, and increased Trails B/Trails A ratios [57–60].

### Methods: Experiment 4

#### Subjects

Thirty-one Veterans with a history of TBI were recruited from the local VANCHCS patient population. The patients included 30 males and one female between the ages of 20 and 61 years (mean age = 35.5 years), with an average of 13.6 years of education (Table 2). All subjects had suffered head injuries and a transient loss or alteration of consciousness, and all were tested at least one year post-injury. Twenty-seven of the patients had suffered one or more combat-related incidents, with a cumulative loss of consciousness of less than 30 min, no hospitalization, and no evidence of brain lesions on clinical MRI scans. These patients were categorized as mild TBI (mTBI). The remaining four patients had suffered more severe accidents with hospitalization, coma duration exceeding eight hours, and post-traumatic amnesia exceeding 72 hrs. These patients were categorized as severe TBI (sTBI). Evidence of posttraumatic stress disorder (PTSD), as reflected in elevated scores on the PCL checklist, was evident in 77% of the TBI sample. Additional patient information is provided in S2 Table. The results were compared with the pooled normative data from 216 controls subjects: i.e., the combined results from Experiment 1 and Experiment 2a.

### Results: Experiment 4

Two TBI patients had been identified as performing with suboptimal effort in previous tests [44, 48, 61], and both showed evidence of malingering on the C-TMT, producing abnormal z-scores on the C-TMT-A and the C-TMT-B, and greater abnormalities on the C-TMT-A than on the C-TMT-B. On the C-TMT-A, the dwell-time/total-time ratios were in the high normal range for both patients. Both subjects were excluded from further comparisons.

Data from the remaining TBI patients (red squares) are shown in the scatter plots in Figs 4–8. Of the entire group [mean z-score = 0.18, F(1,242) = 0.96, NS], only two patients (7.1%), including one with sTBI, showed C-TMT-A abnormalities (Fig 5A), while the rest produced C-TMT-A completion times within the normal range. In contrast, C-TMT-B completion times showed a small but significant increase [regressed z-score = 0.38, F(1,242) = 4.74, p < 0.04, partial ω^2^ = 0.02]. Five patients showed C-TMT-B abnormalities (Fig 5B). All showed greater abnormalities on the C-TMT-B than the C-TMT-A, including three with significantly elevated difference scores (Fig 8).

In addition, movement velocities were slightly slowed in the TBI patient group on both the C-TMT-A [velocity-z = 0.46; F(1,242) = 4.99, p < 0.03, partial ω^2^ = 0.02] and C-TMT-B [velocity-z = 0.54, F(1,242) = 7.71, p < 0.006, partial ω^2^ = 0.03]. Five patients showed abnormal movement velocities on the C-TMT-A (Fig 6A), and six patients showed abnormal velocities on the C-TMT-B (Fig 6B), three of whom also had abnormal C-TMT-A movement velocities. However, comparisons of C-TMT-B minus C-TMT-A completion times and C-TMT-B/C-TMT-A ratios between TBI patients and controls failed to reach significance for either completion times or velocities. We also found no differences between TBI patients and controls in error rates on either test [56].

### Discussion: Experiment 4

Consistent with previous reports of greater Trails B sensitivity to TBI [8, 39, 62], we found small but significant increases in C-TMT-B completion times in TBI patients without significant differences in C-TMT-A completion times. However, as in previous studies of patients with predominantly mild TBI, the effect size of the C-TMT-B abnormalities was small [63], and no significant differences were seen in C-TMT-B versus C-TMT-A difference or ratio measures [42].

Surprisingly, TBI patients showed significant slowing of movement velocities on both the C-TMT-A and the C-TMT-B, similar to that seen in older subjects in Experiment 1 [64]. Small but significant slowing in TBI patients is also seen in other tasks, including finger tapping [46] and choice reaction time [48]. However, some of the impairments, particularly in the mild TBI subjects, may also have reflected the influence of PTSD [65]. Given the small effect sizes, however, these results should be considered more exploratory than definitive. Further studies of larger TBI populations with a greater range of injury severities will be needed to more convincingly establish the sensitivity of the C-TMT to TBI.

Finally, our results underscore the importance of evaluating malingering, particularly in patients with mild TBI [42]. In our group, the inclusion of the two mTBI patients suspected of performing with suboptimal effort would have significantly increased TBI group z-scores, particularly on the C-TMT-A.

## General Discussion

The C-TMT offers several advantages in comparison with the paper-and-pencil versions of the TMT. First, both C-TMT administration and scoring are fully automated, eliminating the influence of the examiner in test-timing and error correction. This makes it more likely that differences in C-TMT results at different sites will reflect differences in subjects, not differences in examiners. Second, the path lengths of the C-TMT-A and C-TMT-B are equated, so that differences in completion time more directly reflect task-specific differences in cognitive processing, independent of path length. Finally, the display is standardized at the beginning of each C-TMT segment, so that neither variations in drawing precision nor the occurrence of errors obfuscate the test pattern. This enables more valid comparisons across different segments of the C-TMT and ensures that test difficulty does not increase over the course of the test among subjects who commit errors or draw imprecisely.

The C-TMT also provides a comprehensive set of metrics, including: (1) Segment-by-segment measures of performance; (2) Separate measures of dwell-time and move-time for each segment and for the test as a whole; (3) Separate measures of error incidence, error time, drawing circuitousness, and drawing velocity; and (4) A complete digital record of paths drawn. These C-TMT metrics permit a more detailed analysis of TMT performance than what is currently possible with manually administered TMTs. For example, C-TMT metrics clarified the effects of aging on completion times: delays in older subjects were due primarily to increases in move-time, as reflected in a reduction of the dwell-time/move-time ratio. Further analysis showed that age-related delays in move-time were most directly associated with reductions in drawing velocity. We found that TMT completion times were also delayed in malingering subjects (Experiment 3) and TBI patients (Experiment 4), but for different reasons. Malingering subjects showed greater increases on the C-TMT-A than on the C-TMT-B, while TBI patients showed the opposite pattern. Moreover, malingering subjects showed an increase in dwell-time/total-time ratios on the C-TMT-A, suggesting that many malingering subjects paused between TMT segments. Finally, the additional C-TMT metrics may add sensitivity to TMT studies. For example, delays in TBI patients on the C-TMT-B were due primarily to reduced drawing velocity, and significant reductions in drawing velocity were also seen on the C-TMT-A without overall abnormalities in completion times. Similarly, C-TMT metrics may help to clarify the nature of TMT delays observed in other patient groups, including patients with schizophrenia [8], Parkinson’s disease [66], mild cognitive impairment [67], and dementia [29]. C-TMT metrics might also elucidate the differences in TMT completion times seen in different ethnic groups [11] and control subjects tested in different countries [9].

### Limitations

Like the standard TMT, the C-TMT also showed substantial learning effects, complicating its use in repeated tests. In addition, C-TMT performance was influenced by computer-use, with completion time delayed in subjects who rarely or never use computers. While more than 90% of the subjects in our Northern California control population used computers for at least 1 hour/day, these subjects had been primarily recruited through Internet advertisements; computer-use may be significantly less widespread in other test populations.

The C-TMT program is available at (www.ebire.org/hcnlab/cognitive-tests/TMT), along with instructions for its use. Excel spreadsheets of subject data are also available on this site. It requires a Windows PC with Presentation software, a computer gaming mouse, and a 17” monitor. The generalization of control norms from the current study may be limited by the high education levels of the subjects in Experiment 1, as well as by non-random subject selection, geographical biases, and other factors.

## Conclusions

A computerized version of the TMT (C-TMT) was shown to increase the precision and reproducibility of TMT assessment. The C-TMT provides segment-by-segment performance analyses of drawing path, dwell-time, move-time, and error-time, and permits the further analysis of move-time components including drawing circuitousness and velocity. Completion times on both the C-TMT-A and C-TMT-B were strongly influenced by age, due primarily to age-related reductions in drawing velocity. Significant learning effects were found with repeated test administration. Nearly half of the subjects instructed to malinger showed abnormal completion times on the C-TMT-A, while a smaller percentage showed abnormalities on the C-TMT-B. Malingering subjects with abnormal performance could be distinguished from control subjects and TBI patients with abnormally slowed completion times due to (1) greater deficits on the C-TMT-A than the C-TMT-B, and (2) a tendency to pause on circles during C-TMT-A testing, increasing the dwell-time/total-time ratio. TBI patients produced slowed completion times on the C-TMT-B and reduced movement velocities on both the C-TMT-A and C-TMT-B. The C-TMT improves the reliability and sensitivity of the trail making test of processing speed and executive function.

## Supporting Information

S1 Table Segment-by-segment performance on Trails A and B. (DOCX)

S2 Table TBI patient characteristics. (DOCX)
