# Supplementary material for: The Effects of Aging, Malingering, and Traumatic Brain Injury on Computerized Trail-Making Test Performance
Source: PLoS One. 2015 Jun 10;10(6):e0124345. doi: 10.1371/journal.pone.0124345 (PMC4465490; doi:10.1371/journal.pone.0124345)
Supplement: S1 Table — (DOCX) [file pone.0124345.s001.docx]

| **Table S1. Segment-by-segment performance on Trails A and B.** | | | | | | |
| --- | --- | --- | --- | --- | --- | --- |
| **TRAILS A** | | | | | | |
| Circle | Length | Time | Dwell | Move | Circuit | Vel |
| 2 | 144 | 2135 | 622 | 1512 | 1.76 | 0.20 |
| 3 | 206 | 2564 | 692 | 1872 | 1.62 | 0.26 |
| 4 | 247 | 2513 | 595 | 1919 | 1.47 | 0.30 |
| 5 | 260 | 2564 | 763 | 1801 | 1.50 | 0.30 |
| 6 | 269 | 1518 | 514 | 1004 | 1.21 | 0.38 |
| 7 | 83 | 1163 | 331 | 833 | 1.68 | 0.19 |
| 8 | 360 | 1466 | 371 | 1095 | 1.14 | 0.41 |
| 9 | 111 | 938 | 274 | 664 | 1.39 | 0.26 |
| 10 | 209 | 1414 | 295 | 1120 | 1.29 | 0.29 |
| 11 | 255 | 1290 | 312 | 978 | 1.19 | 0.34 |
| 12 | 99 | 898 | 276 | 622 | 1.43 | 0.25 |
| 13 | 171 | 1115 | 293 | 822 | 1.23 | 0.29 |
| 14 | 144 | 1046 | 295 | 750 | 1.31 | 0.27 |
| 15 | 180 | 1093 | 318 | 776 | 1.22 | 0.31 |
| 16 | 98 | 922 | 265 | 657 | 1.29 | 0.22 |
| 17 | 341 | 1889 | 413 | 1476 | 1.26 | 0.33 |
| 18 | 205 | 1171 | 308 | 863 | 1.20 | 0.32 |
| 19 | 95 | 1174 | 358 | 816 | 1.81 | 0.23 |
| 20 | 317 | 1916 | 463 | 1453 | 1.46 | 0.34 |
| 21 | 358 | 1784 | 428 | 1356 | 1.20 | 0.37 |
| 22 | 270 | 1435 | 357 | 1078 | 1.30 | 0.37 |
| 23 | 368 | 1628 | 358 | 1270 | 1.17 | 0.39 |
| 24 | 203 | 1203 | 303 | 901 | 1.30 | 0.34 |
| 25 | 175 | 1171 | 267 | 904 | 1.19 | 0.31 |
| TRAILS B | | | | | | |
|  | Length | Time | Dwell | Move | Circuit | Vel |
| A | 137 | 1538 | 354 | 1184 | 1.32 | 0.20 |
| 2 | 228 | 2577 | 1031 | 1546 | 1.52 | 0.29 |
| B | 139 | 1416 | 369 | 1048 | 1.35 | 0.23 |
| 3 | 237 | 2219 | 754 | 1464 | 1.38 | 0.30 |
| C | 154 | 1560 | 487 | 1073 | 1.39 | 0.25 |
| 4 | 175 | 1884 | 440 | 1444 | 1.29 | 0.20 |
| D | 295 | 3714 | 1373 | 2341 | 1.38 | 0.25 |
| 5 | 164 | 2864 | 1038 | 1826 | 1.64 | 0.21 |
| E | 250 | 3994 | 1210 | 2784 | 1.52 | 0.26 |
| 6 | 155 | 2322 | 697 | 1625 | 1.45 | 0.21 |
| F | 140 | 2086 | 721 | 1365 | 1.57 | 0.21 |
| 7 | 340 | 2929 | 743 | 2186 | 1.36 | 0.29 |
| G | 136 | 1521 | 436 | 1085 | 1.35 | 0.23 |
| 8 | 236 | 2890 | 780 | 2110 | 1.68 | 0.23 |
| H | 385 | 2523 | 647 | 1875 | 1.23 | 0.33 |
| 9 | 201 | 2162 | 572 | 1591 | 1.36 | 0.25 |
| I | 407 | 3393 | 656 | 2737 | 1.40 | 0.33 |
| 10 | 139 | 2384 | 628 | 1756 | 1.73 | 0.20 |
| J | 104 | 1522 | 474 | 1048 | 1.49 | 0.19 |
| 11 | 394 | 4188 | 1045 | 3142 | 1.51 | 0.22 |
| K | 276 | 2447 | 569 | 1878 | 1.26 | 0.27 |
| 12 | 77 | 2340 | 745 | 1595 | 2.59 | 0.17 |
| L | 212 | 1757 | 360 | 1397 | 1.28 | 0.31 |
| 13 | 188 | 1061 | 174 | 887 | 1.13 | 0.28 |
| For each segment, the length of the segment (in pixels), and the means of completion time, dwell- time, move-time (in ms), circuitousness (Circuit), and velocity (Vel, in pixels/ms). Data from Experiment 1. | | | | | | |
