# Supplementary material for: The Effects of Aging, Malingering, and Traumatic Brain Injury on Computerized Trail-Making Test Performance
Source: PLoS One. 2015 Jun 10;10(6):e0124345. doi: 10.1371/journal.pone.0124345 (PMC4465490; doi:10.1371/journal.pone.0124345)
Supplement: S2 Table — (DOCX) [file pone.0124345.s002.docx]

| **Table S2. TBI patient characteristics**. | | | | | |
| --- | --- | --- | --- | --- | --- |
| **ID** | **Age** | **EDU** | **Etiology** | **TBI** | **PCL** |
| **P29^c^** | 35 | 12 | MVA | Severe | 59 |
| **P28^c,d^** | 24 | 12 | Blast | Mild | 54 |
| **P27^c,d^** | 28 | 12 | Blast | Mild | 66 |
| **P26^d^** | 46 | 12 | MVA | Severe | 42 |
| **P24^c,d^** | 57 | 14 | MVA | Severe | 56 |
| **P22** | 30 | 14 | MVA | Mild | -- |
| **P21^c^** | 52 | 18 | MVA | Mild | 27 |
| **P20^c,d^** | 41 | 14 | Blast^a^ | Mild | 45 |
| **P19** | 20 | 14 | Blast^a^ | Mild | 41 |
| **P18^b,c^** | 46 | 14 | MVA | Severe | 46 |
| **P17^d^** | 25 | 16 | Fall | Mild | 0 |
| **P16** | 28 | 13 | Blast | Mild | 47 |
| **P15^d^** | 25 | 14 | Blast | Mild | 57 |
| **P14** | 29 | 10 | Blast | Mild | 54 |
| **P13^c,d^** | 47 | 14 | Blast^a^ | Mild | 52 |
| **P12** | 28 | 12 | Fall | Mild | 43 |
| **P11^c^** | 29 | 14 | Blast | Mild | 27 |
| **P10^d^** | 61 | 18 | MVA^a^ | Mild | 52 |
| **P09 ^c,d^** | 27 | 15 | Blast | Mild | 72 |
| **P08 ^c,d^** | 48 | 13 | Blast | Mild | 59 |
| **P06^c^** | 49 | 12 | Fall | Mild | 47 |
| **P05^c^** | 28 | 14 | Fall | Mild | 68 |
| **P04^c,d^** | 39 | 13 | MVA | Mild | 64 |
| **P03^c,d^** | 25 | 12 | Blast^a^ | Mild | 72 |
| **P02 ^c,d^** | 23 | 14 | Fall | Mild | 67 |
| **P01 ^c,d^** | 29 | 14 | Fall | Mild | 47 |
| TBI=traumatic brain injury severity. PCL=PTSD Checklist. Age in years. EDU=years of education. MVA=moving vehicle accident. ^a^Multiple TBIs. ^b^Female. ^c^Chronic Pain. ^d^Sleep Problems. | | | | | |
